# Supplementary material for: FlpStop, a tool for conditional gene control in Drosophila
Source: eLife. 2017 Feb 17;6:e22279. doi: 10.7554/eLife.22279 (PMC5342825; doi:10.7554/eLife.22279)
Supplement: Figure 3—source data 1. — DOI: http://dx.doi.org/10.7554/eLife.22279.010 [file elife-22279-fig3-data1.docx]

**Figure 3 – Source Data 1** Table of exact p-values and statistical tests

| **a** *Gad1* lethality (one-proportion z-test) |  |
| --- | --- |
|  | Predicted survival 33% |
| Null 1 / Null 2 | < 0.0001 |
| D / Null 1 | < 0.0001 |
| D / Null 2 | < 0.0001 |
| ND / Null 1 | 0.6928 |
| ND / Null 2 | 0.1932 |
|  |  |
| **b** *Rdl* lethality (one-proportion z-test) |  |
|  | Predicted survival 33% |
| Null 1 / Null 2 | < 0.0001 |
| D / Null 1 | < 0.0001 |
| D / Null 2 | < 0.0001 |
| ND / Null 1 | 0.0001 |
| ND / Null 2 | 0.0752 |
|  |  |
| **c** *para* paralysis (two-tailed Fisher’s exact test) |  |
|  | TS / + (33**°**C or 35**°**C) |
| TS / TS (33**°**C) | 1.95E-6 |
| TS / TS (35**°**C) | 2.30E-19 |
| D / TS (33**°**C) | 8.70E-12 |
| D / TS (35**°**C) | 3.08E-13 |
| ND / TS (33**°**C) | 1 |
| ND / TS (35**°**C) | 1 |
|  |  |
| **d** *cac* paralysis (two-tailed Fisher’s exact test) |  |
|  | TS / + (37**°**C or 40**°**C) |
| TS / TS (37**°**C) | 4.00E-5 |
| TS / TS (40**°**C) | 4.28E-17 |
| D / TS (37**°**C) | 1.70E-14 |
| D / TS (40**°**C) | 2.63E-26 |
| ND / TS (37**°**C) | 1 |
| ND / TS (40**°**C) | 1 |
|  |  |
| **f** *cac* q-RT-PCR (unpaired two-tailed Student’s t-test) |  |
|  | ND / + |
| D / + | 0.0214 |
|  |  |
| **g** *Gad1* q-RT-PCR (unpaired two-tailed Student’s t-test) |  |
|  | ND / + |
| D / + | 0.0032 |
|  |  |
| **h** *para* q-RT-PCR (unpaired two-tailed Student’s t-test) |  |
|  | ND / + |
| D / + | 0.0334 |
| **i** *Rdl* q-RT-PCR (unpaired two-tailed Student’s t-test) |  |
|  | ND / + |
| D / + | 0.0048 |

Figure 3 –figure supplement 1:

| **a** *ChAT* lethality (one-proportion z-test) |  |
| --- | --- |
|  | Predicted survival 33% |
| Null 1 / Null 2 | < 0.0001 |
| D / Null 1 | 0.4318 |
| D / Null 2 | 0.0298 |
| ND / Null 1 | 0.5126 |
| ND / Null 2 | 0.0807 |
|  |  |
| **b** *VGlut* lethality (one-proportion z-test) |  |
|  | Predicted survival 33% |
| Null 1 / Null 1 | < 0.0001 |
| D / Null 1 | < 0.0001 |
| ND / Null 1 | 0.7389 |

| **c** *Shal* q-RT-PCR (unpaired two-tailed Student’s t-test) |  |
| --- | --- |
|  | ND / ND |
| D / D | 0.0219 |
