# Supplementary material for: FlpStop, a tool for conditional gene control in Drosophila
Source: eLife. 2017 Feb 17;6:e22279. doi: 10.7554/eLife.22279 (PMC5342825; doi:10.7554/eLife.22279)
Supplement: Figure 7—source data 1. — All statistical tests were two-sample Student’s t-tests, Bonferroni correction for multiple comparisons. DOI: http://dx.doi.org/10.7554/eLife.22279.018 [file elife-22279-fig7-data1.docx]

**Figure 7 – Source Data 1** Table of exact p-values

| **a** Cell Body |  |
| --- | --- |
|  | Experimental |
| No Flp control | 3.73E-03 |
| Heterozygous control | 5.51E-04 |
|  |  |
| **b** Layer M1 |  |
|  | Experimental |
| No Flp control | 1.06E-01 |
| Heterozygous control | 1.82E-09 |
|  |  |
| **c** Layer M5 |  |
|  | Experimental |
| No Flp control | 9.56E-10 |
| Heterozygous control | 9.56E-10 |
|  |  |
| **d** Layer M8 |  |
|  | Experimental |
| No Flp control | 3.15E-02 |
| Heterozygous control | 9.90E-01 |
|  |  |
| **e** Layer M10 |  |
|  | Experimental |
| No Flp control | 7.03E-01 |
| Heterozygous control | 9.47E-02 |
|  |  |
| **f** Layer Lo1 |  |
|  | Experimental |
| No Flp control | 1.74E-04 |
| Heterozygous control | 1.37E-08 |
|  |  |
| **g** Layer Lo3 |  |
|  | Experimental |
| No Flp control | 6.61E-09 |
| Heterozygous control | 1.39E-09 |
